# The Effect of Mitomycin C on Induction of Shiga Toxin Production in Clinical STEC Isolates

**DOI:** 10.3390/toxins17060267

**Published:** 2025-05-27

**Authors:** Surangi H. Thilakarathna, Brendon Parsons, Linda Chui

**Affiliations:** 1 Department of Laboratory Medicine and Pathology, Faculty of Medicine and Dentistry, University of Alberta, Edmonton, AB T6G 1C9, Canada; sthilaka@ualberta.ca (S.H.T.); brendon.parsons@albertaprecisionlabs.ca (B.P.); 2 Alberta Precision Laboratories-Public Health Laboratory (ProvLab), Edmonton, AB T6G 2J2, Canada

**Keywords:** Shiga toxin-producing *Escherichia coli* (STEC), mitomycin C, enzymatic immunoassays (EIA), STEC subtypes, Shiga toxin production, diagnostics

## Abstract

Early determination of the Shiga toxin type of Shiga toxin-producing *Escherichia coli* (STEC) is crucial for guiding STEC-infected patients for proper and timely treatment and patient care. Most diagnostic microbiology laboratories rely on PCR assays to detect the presence of *stx1* and/or *stx2* and enzymatic immunoassays (EIA) to detect the presence of the Shiga toxins 1 and/or 2 in STEC-positive stool samples. Occasionally, the stool samples test positive for STEC by PCR assays but test negative for the presence of Shiga toxins. Insufficient toxin production under laboratory conditions is the main culprit of this discordance. To test whether EIA-based STEC detection could be improved, various clinical STEC strains were treated with mitomycin C, which is a commonly used inducer of Shiga toxin production. A dose-dependent increase in Shiga toxin production, in response to mitomycin C doses of up to 500 ng/mL, was observed without any bactericidal effects. Depending on the serotype, 5–50 times more Shiga toxin 2 was produced than Shiga toxin 1. Shiga toxin production was not induced by the mitomycin C treatment in certain STEC serotypes carrying the toxin subtypes *stx1a*, *stx2a*, *2b*, *2f*, *or 2h*. This diversity in toxin production indicates that other factors may determine toxin expression in certain STEC strains, which warrant further exploration.

## 1. Introduction

Shiga toxin-producing *Escherichia coli* (STEC) is a Gram-negative gastroenteric bacterial pathogen that can cause typical acute gastroenteritis symptoms like diarrhea, vomiting, fever, and stomach pain [1]. However, some STEC strains are highly virulent and can cause severe disease outcomes [1]. Generally, STEC strains only expressing *stx1* are associated with mild disease or asymptomatic carriage, while strains expressing both *stx1* and *stx2* are associated with greater pathogenicity, and strains expressing *stx2* alone are recognized as ‘high risk’ due to their association with severe disease outcomes such as hemolytic uremic syndrome (HUS) and bloody diarrhea [2,3]. These highly virulent *stx2* STEC strains need to be managed cautiously, as administration of antibiotics to patients infected with *stx2* STEC strains have been linked with adverse reactions and worsened severe symptoms leading to kidney damage and even death [4]. Therefore, identifying the Shiga toxin type early in the diagnosis is crucial for STEC infection management and guiding patients for proper treatments and care.

Frontline diagnostic laboratories in Alberta use BD MAX^TM^ (BD Life Sciences, Sparks, MD, USA), a multi-analyte nucleic acid amplification platform, to detect the presence of enteric pathogens in patient stool specimens. The BD MAX^TM^ enteric bacterial panel includes specifically designed sets of primers and probes for *Salmonella* spp., *Shigella* spp./enteroinvasive *Escherichia coli* (EIEC), *Campylobacter* spp., and STEC [5,6]. The STEC primers and probes used in the BD MAX^TM^ panel are designed to detect the *stx1/stx2* genes but without any differentiation between them [5]. For further differentiation between the two toxins, laboratories can perform specific PCR assays or enzyme immunoassays (EIA). The SHIGA TOXIN QUIK CHEK^TM^ test is a qualitative EIA kit developed by TECHLAB, Inc. (Blacksburg, VA, USA) and is widely used in Canadian microbiology laboratories. Using monoclonal and polyclonal antibodies, this EIA can detect the presence of Shiga toxin 1 and Shiga toxin 2 and differentiate one from the other [7]. However, there are instances in which a stool sample tests positive for STEC by BD MAX^TM^, but negative for the presence of Shiga toxins using these EIA kits (personal communication). These contradictory results bring challenges to the frontline laboratories when reporting STEC infections. In such a scenario in Alberta, these stool samples are forwarded to Alberta Precision Laboratories-Provincial Laboratory for Public Health (ProvLab) for further investigations. This additional testing increases the costs and turnaround time for reporting.

A negative EIA result could be due to numerous reasons, such as a low bacterial load resulting in the reduction of toxin level or decreased expression of the *stx* gene in STEC below the detection level of the assay. If a limited *stx* expression in the stool failed to be detected by the EIA, the addition of an inducer can potentially enhance the Shiga toxin production. Application of antibiotics as enhancers to induce Shiga toxin production under laboratory settings has been previously suggested [7,8,9]. Antibiotic treatment is generally not recommended for patients with STEC infections, as certain antibiotics can induce Shiga toxin overproduction as a response to the triggered host SOS response [3,10]. Although this is an extremely critical condition in the clinical setting, it can be advantageous in a diagnostic setting to improve test methods such as EIA. Mitomycin C is one of the antibiotics most commonly used to induce Shiga toxin production in a laboratory setting. However, the use of mitomycin C in a clinical setting to induce Shiga toxin production in STEC isolates or STEC-positive stool samples has not been previously reported. Therefore, the objective of this study was to evaluate the effect of mitomycin C on the enhancement of Shiga toxin production by clinically relevant STEC isolates in broth and in stool samples.

## 2. Results

### 2.1. Shiga Toxin Production by Three STEC Isolates Treated with Different Concentrations of Mitomycin C

To assess the effect of mitomycin C on Shiga toxin induction, three STEC isolates with distinct Shiga toxin gene profiles were exposed to increasing concentrations of the antibiotic in trypticase soy broth (TSB), incubated overnight, and the toxin production was subsequently measured. Shiga toxin production in the three tested STEC isolates increased with the increasing mitomycin C concentrations in a dose-dependent manner (Table 1). The highest mitomycin C concentration, which was 500 ng/mL, induced the production of the highest amount of Shiga toxin in the tested STEC isolates (Supplementary Figure S1, *p* < 0.05). Real-time PCR Ct values for all tested mitomycin C concentrations stayed consistent at ~13 Ct for O5:H19. For O26:H11 and O157:H7, the Ct values ranged from ~8–12 and decreased by ~1–3 Ct when treated with >10 ng/mL mitomycin C. These results showed that the Shiga toxin production was induced after the overnight incubation with the mitomycin C treatment. There was a difference between the production of Shiga toxin 1 and 2, as Shiga toxin 2 was increased 5–50 times more than Shiga toxin 1 after the mitomycin C treatment. Compared to the O157 *stx1/2* strain, the O26 strain was a high toxin producer, where ~10 times more Shiga toxin 2 was produced by O26:H11 treated with 500 ng/mL mitomycin C.

### 2.2. Shiga Toxin Production by Clinical STEC Isolates Treated with 500 ng/mL Mitomycin C

To evaluate Shiga toxin induction in other clinical STEC isolates by the optimum mitomycin C concentration (500 ng/mL), 53 clinical STEC isolates were enriched overnight in TSB with and without the mitomycin C treatment, and their growth and toxin production were assessed. The PCR Ct values for the non-treated and the mitomycin C-treated broths were similar for ~80% of the isolates, where ∆Ct was <1 (Table 2). This was an indication that the mitomycin C treatment did not significantly affect the growth of the majority of isolates. Toxin quantification was not performed for these samples.

Shiga toxin production was not detected by the SHIGA TOXIN QUIK CHEK^TM^ in ~17% (9/53) of the isolates under non-treated conditions (Table 2). After an overnight enrichment with mitomycin C, 4/9 of isolates (~44%) produced sufficient amounts of toxin to be detected by EIA. The mitomycin C treatment did not enhance the toxin production of the other five isolates that showed negative EIA results. These five STEC isolates belonged to different serotypes (O26:H11 *stx2a*, O156:H14 *stx2f/h/b*, O157:H7 *stx2a*, O17/O77/O44/O106:H45 *stx2d*, and O2/O50:H7 *stx2f/h/b*), indicating lack of a direct relationship between the serotype or the toxin subtype and the limited toxin production. Two other STEC isolates (O166:H15 and O85:H1) with the *stx2f/h/b* toxin subtype were weakly detected by EIA with and without the mitomycin C treatment. It is also possible that these *stx2* toxin subtypes are not effective antigenic responders. Supplementary Figure S2 shows improved SHIGA TOXIN QUIK CHEK^TM^ results after the mitomycin C treatment and results for high and low Shiga toxin-producing STEC isolates.

### 2.3. Shiga Toxin Production by STEC in Clinical Stool Samples Treated with 500 ng/mL Mitomycin C

To evaluate the impact of enrichment media and mitomycin C treatment on Shiga toxin detection from clinical stool samples, 15 STEC-positive patient-stool samples were analyzed for toxin gene profiles, growth performance, and toxin expression across two culture media: TSB and Gram-negative broth (GNB). From the tested stool samples, 7/15 were *stx1*-postive, 3/15 were *stx2*-positive, and 5/15 were *stx1/2*-positive. Based on the PCR results, GNB was the better enrichment medium for both non-treated and mitomycin C-treated stool samples (Ct values: TSB > GNB, Table 3). Only two stool samples with O157:H7 *stx1a/stx2a* (ID #2) and O26:H11 *stx1a* (ID #5) showed better growth in TSB under non-treated or mitomycin C-treated conditions (Table 3). Considering potential sampling error, TSB-GNB < −1 was not considered as a treatment effect.

The average amount of toxin produced across all STEC stool samples enriched in TSB with and without the mitomycin C treatment was statistically similar (TSB_NT_ vs. TSB_MC_; *p* > 0.05). On the other hand, STEC stool samples enriched in GNB produced a significantly higher amount of toxin with the mitomycin C treatment compared to the non-treated samples (*p* = 0.0077). Among the two media, GNB trended toward producing more Shiga toxin compared to TSB with and without the mitomycin C treatment (mean ± SD for the amount of toxin produced (Shiga toxin 1 and 2 combined, ng/mL) by each treatment: TSB_NT_: 0.49 ± 0.58, TSB_MC_: 1.67 ± 2.77, GNB_NT_: 1.06 ± 1.46, GNB_MC_: 3.34 ± 5.16). STEC with *stx2* in clinical stool samples trended to produce more Shiga toxins compared to STEC with *stx1* (Table 4), although a statistically significant difference was not observed.

Overall, 5/15 stool samples (~33%) showed real-time PCR-positive but SHIGA TOXIN QUIK CHEK^TM^-negative results for both non-treated and mitomycin C-treated conditions in both broths (Supplementary Figure S3). From those five stool samples, two had Ct values between 13–19, indicating bacterial loads of ~10^6^–10^8^ CFU/mL based on our preliminary results (unpublished data). Therefore, even with low Ct values that pointed to higher bacterial loads, some serotypes, such as O128:H2/O5:H9 *stx1/2*, O9:H7 *stx2*, O151/O118:H16 *stx1*, O103:H2 *stx1*, and O26:H11 *stx1*, produced very low amounts of toxins that were not detected by either EIA, which indicates a need for further investigation.

## 3. Discussion

PCR and EIA are assays commonly used in diagnostic laboratories to detect STEC in stool samples [11,12]. While PCR assays are designed to detect the STEC DNA, EIAs are designed to detect the Shiga toxins produced by STEC [11]. Therefore, in certain situations, the PCR and EIA results might not corroborate. For an example, under laboratory conditions, a PCR-positive stool sample can show a negative EIA result. This is an indication that although STEC is present in the stool sample, the expression of *stx* is at a suboptimal level. In such scenarios, Shiga toxin induction can be highly beneficial to produce a sufficient level of Shiga toxin to obtain positive EIA results. A large number of studies are available on Shiga toxin induction by various inducers, including antibiotics, to investigate clinical outcomes in patients [4,8,13,14,15]. However, studies performed on in vitro Shiga toxin induction for the purpose of STEC detection are limited. The current study evaluated the ability of mitomycin C treatment to improve EIA results under laboratory conditions for effective STEC detection.

This study evaluated Shiga toxin production by STEC after an overnight enrichment with mitomycin C in culture broths. Three STEC isolates that represented *stx1*, *stx2*, and *stx1*/*2* were selected for optimizing the mitomycin concentration to use in the experiments. We observed that overall, Shiga toxin production by *stx1* STEC isolates was low compared to *stx2* STEC isolates. The difference was more apparent with increasing mitomycin C concentrations. Our findings are in agreement with previous reports where mitomycin C induced the production of more Shiga toxin 2 than Shiga toxin 1 [16]. Shiga toxins 1 and 2 are induced by different induction methods [16]. Phage-inducing agents like mitomycin C induce the expression of *stx2* and activate Shiga toxin 2 production, while low iron conditions activate the production of Shiga toxin 1 [10,16]. Therefore, when considering enhancing Shiga toxin production in a clinical laboratory setting for STEC detection, methods that are effective in inducing both Shiga toxins need to be carefully considered.

STEC growth and toxin production can depend on the culture media used. Preliminary experiments carried out to investigate the growth of the three STEC isolates (O5:H19 *stx1*, O26:H11 *stx2*, and O157:H7 *stx1/2*) in TSB and GNB showed that the isolates in pure culture preferred TSB over GNB, which was in agreement with a previous study [17]. GNB selectively enhances the growth of Gram-negative bacteria in a heterogeneous mixture of Gram-positive and Gram-negative bacteria. Since the bacterial load inoculated into the 3 mL broth was ~100 CFU/mL in the current study and no other competing organisms were present in the broth, it appeared that the growth of the STEC isolates was not well supported by GNB TSB, on the other hand, is a general medium and supported STEC isolate growth. Therefore, TSB was selected as the enrichment medium for all experiments involving pure STEC isolates (in broth media). Since different diagnostic microbiology laboratories use either GNB or TSB for overnight stool enrichment, both TSB and GNB were tested for STEC-positive stool enrichment. According to the results, STEC growth in stool samples was better supported by GNB compared to TSB, but toxin production was similar in both TSB and GNB. Random sampling error may influence the findings; however, using 10% stool suspensions in the experiments would help minimize this error. A previous study reported better STEC growth in TSB when spiked in STEC-negative stool samples, but Shiga toxin production was not assessed in that study, preventing comparisons with our findings [17]. It is possible that stool microbiome in STEC-positive stool samples in our study contributed to the observed differences from the previous study. Contrasting with our findings, a study reported that Shiga toxin production depended on the type of broth used to culture STEC [18]. Among the eight broths tested by the authors, TSB resulted in low toxin production compared to the better-performing *E. coli* broth [18]. The results cannot be directly compared with our findings, as GNB was not tested in that study.

The *stx* expression mainly depends on prophage induction [19]. Numerous antibiotics have been tested for their prophage induction ability on STEC [15,20]. Antibiotics such as gentamicin, azithromycin, and meropenem did not induce Shiga toxin production in highly virulent *stx2a* STEC [20]. On the other hand, mitomycin C is a commonly used prophage-inducing antimicrobial [14,20]. Due to its potent effect, it has been used as a positive control to test the toxin induction ability of other antibiotics [20]. Mitomycin C can trigger the host SOS response by releasing H_2_O_2_ and damaging host DNA through the promotion of cross-linking events, alkylation, DNA strand breakage [14], and activation of RecA (a regulator of bacterial SOS response) [19], leading to lytic phage activation and Shiga toxin production [19]. Recovery or cell death from the DNA damage is dependent on the released H_2_O_2_ dose, as a low H_2_O_2_ dose can promote DNA repair [14]. Therefore, both the STEC growth and toxin expression can be promoted by the appropriate mitomycin C concentration. Previous studies have used mitomycin C concentrations ranging from 50 ng/mL to 50 μg/mL [9,14,20]. We observed concentration-dependent Shiga toxin production when mitomycin C was applied at 5–500 ng/mL. We tested the mitomycin C response of a limited number of STEC isolates, which is a limitation of this study. Therefore, it is challenging to generalize the optimum mitomycin C concentration or the effective range of concentrations across all STEC strains and/or *stx* subtypes.

Mitomycin C has been used in previous studies to induce Shiga toxin production in STEC subtypes. Mitomycin C increased the toxin production in different clinical STEC strains with *stx1a*, *stx2a*, *2c*, *2d* [21], and non-human STEC strains with *stx2e* subtypes [22]. From the STEC isolates tested in the current study, there was one O25:H1 with the *stx2e* subtype. In agreement with the previous findings [22], this particular STEC did not show toxin production when non-treated, but showed positive SHIGA TOXIN QUIK CHEK^TM^ results after treatment with mitomycin C. Mitomycin C reportedly increased Shiga toxin 2 production in the *stx2a* subtype of 12 highly virulent STEC serotypes [20]. In the current study, ~20% of STEC isolates (pure culture) and ~33% of STEC strains in stool samples carried the *stx2a* subtype. However, not all of these STEC isolates with *stx2a* responded the same to the mitomycin C treatment. Shiga toxin production was not improved in O157:H7, O26:H11 (Table 2, STEC isolates in culture), or O9:H7 (Table 4, STEC in stool), after the mitomycin C treatment. Apart from *stx2a*, other serotypes with *stx2d* and *stx2f/h/b* subtypes were also not affected by the mitomycin C treatment. For example, O26:H11 with *stx2a*, O17/O77/O44/O106:H45 with *stx2d*, O2/O50:H7, and O156:H14 with *stx2f/h/b* did not produce detectable levels of toxins after the mitomycin C treatment. The selective induction of Shiga toxin production by mitomycin C in certain STEC serotypes and/or subtypes, but not in others, highlights the need for further investigation into the underlying mechanisms. Some STEC strains with a higher basal level of Shiga toxin expression are known to be less responsive to inducers such as mitomycin C [21]. This might not be directly relevant to the STEC isolates that were unaffected by the mitomycin C treatment in the current study, as they showed negative EIA results when non-treated. More STEC serotypes and subtypes need to be tested to better understand the mitomycin C-induced Shiga toxin production by STEC.

The current study used primers and probes developed to detect *stx* gene in STEC [1,23]. Specifically, the TaqMan probes used in the real-time PCR assay differentiated the *stx1* from *stx2* [23]. Therefore, using these sets of real-time PCR primers and probes was an added advantage, as it served as a secondary assay that confirmed the EIA results. Further, when no Shiga toxins were detected by the EIA for PCR-positive samples, the secondary real-time PCR results were beneficial to confirm positive bacterial growth with either no toxin production or inability of the EIA to detect the produced toxin. The small STEC-positive stool sample size is a major limitation in our study. A larger sample size would be beneficial to minimize the effect from the observed variability in Shiga toxin production. Further, we tested only 53 STEC isolates (pure culture). Due to resource limitations, only the presence of Shiga toxin was determined for these 53 STEC isolates, without quantification by the RUO Quantitative Shiga toxin ELISA assay. This is a limitation of the study, as the amount of toxin produced was not determined for comparison with the toxin produced by different STEC serotypes and/or subtypes. To address the limitations, a more comprehensive study testing more STEC serotypes and *stx* subtypes and a larger number of stool samples needs to be performed.

## 4. Conclusions

This study focused on using mitomycin C, a commonly used antibiotic, as an enhancer to induce the production of Shiga toxin in clinical STEC isolates. Most of the tested STEC isolates positively responded to the mitomycin C treatment. As Shiga toxin 2 was enhanced more by the mitomycin C treatment than Shiga toxin 1, it is ideal to consider methods suitable for both *stx1* and *stx2* induction. However, some STEC isolates responded poorly to mitomycin C regardless of the *stx* type. At this point, a clear correlation between mitomycin-induced Shiga toxin production and the STEC serotype or subtype was not observed. More research is warranted to understand the effects of antibiotics on Shiga toxin induction by STEC in order to consider antibiotics as enhancers of Shiga toxin production in a diagnostic laboratory setting.

## 5. Materials and Methods

### 5.1. STEC Isolates and Growth Conditions

Clinical strains of STEC frozen in skim milk were obtained from Alberta Precision Laboratories-Provincial Laboratory for Public Health (ProvLab). Upon receipt, the isolates were plated on sheep blood agar plates (BAP, Dalynn Biologicals, Calgary, AB, Canada) and were incubated overnight at 37 °C. On the following day, a single colony from each BAP was inoculated separately into TSB (BBL™ Trypticase™ Soy Broth, Becton, Dickinson and Company, Sparks, MD, USA) and incubated at 37 °C with moderate shaking until the OD value reached ~0.5 (for ~3–4 h) (MicroScan Turbidity Meter, Siemens Healthcare Diagnostics Limited, Los Angeles, CA, USA). Our preliminary data showed a 10^−6^ dilution prepared from an enriched broth culture with a 0.5 OD was equivalent to ~1000 CFU/mL; therefore, 10^−6^ dilution was selected for the mitomycin C treatments. Using each enriched broth culture, 10-fold serial dilutions from neat to 10^−6^ were prepared in phosphate saline buffer (PBS), and 100 μL of 10^−6^ dilution was used in experiments as described in 5.5. Three clinical STEC isolates with different serotypes and *stx* types were selected to perform the experiments for determining the optimal mitomycin C concentration. They were O5:H19 (*stx1c*), O26:H11 (*stx2a*), and O157:H7 (*stx1a* and *stx2a*).

### 5.2. Nucleic Acid Extraction

A 100 μL aliquot from each mitomycin C-treated and non-treated enriched broth culture was centrifuged at 17,115× *g* for 5 min. The supernatant was removed, and the cell pellets were resuspended in 100 μL of rapid lysis buffer (RLB; 100 mM NaCl, 10 mM Tris-HCL pH 8.3, 1 mM EDTA pH 9.0, 1% Triton X-100), then heated at 95 °C for 15 min. The suspension was centrifuged at 17,115× *g* for 5 min, and 5 μL of the supernatant was used as the template for the real-time PCR.

### 5.3. Real-Time PCR Assay Conditions

The in-house primer-probe (Integrated DNA Technologies, IDT, Skokie, IL, USA) set that targeted a conserved region of the *stx* gene [23] was used in the real-time PCR assays. Primers and probes used in the study are listed in Table 5.

The real-time PCR mixture was prepared by combining 10 μL of 2X PrimeTime^®^ Gene Expression Master mix (Integrated DNA Technologies, IDT, Skokie, IL, USA), 2 μL of nuclease-free water (Invitrogen™, Live Technologies, Rochester, NY, USA), 3 μL of the in-house primer-probe mixture (0.22 μM final concentration of the probe, 0.33 μM final concentration of each of the primers), and 5 μL of the DNA template to contain a final reaction volume of 20 μL. Positive DNA for the *stx* gene and a no-template control (nuclease-free water) were included in each run. Amplification conditions consisted of 95 °C for 3 min followed by 40 cycles of 95 °C for 5 s and 60 °C for 30 s on the 7500 FAST real-time PCR system (Applied Biosystems, Foster City, CA, USA). The *stx* subtypes were identified based on the predictions from Whole Genome Sequencing data using the STECFinder software version 1.1.1 (https://github.com/LanLab/STECFinder, accessed on 24 April 2025).

### 5.4. Enzymatic Immunoassays for Shiga Toxin Detection and Quantification

The presence of the Shiga toxin in the overnight-enriched TSB broth cultures was qualitatively determined using the SHIGA TOXIN QUIK CHEK^TM^ kits as per manufacturer’s instruction. Into 650 μL of the provided diluent, 100 μL of the enriched broth and one drop of the provided conjugate were added. After thoroughly vortexing, 500 μL of the mixture was added to the SHIGA TOXIN QUIK CHEK^TM^ cassette. After a 15 min incubation at room temperature, the reaction window was washed with the wash buffer. After adding the substrate and incubating for 10 min at room temperature, the results were visually observed and recorded.

The Shiga toxin was further quantified using the Research Use Only (RUO) Quantitative Shiga toxin ELISA kits (TECHLAB, Inc., Blacksburg, VA, USA) following the instructions provided by TECHLAB, Inc. The Quantitative Shiga toxin ELISA kits consisted of separate microplates for Shiga toxins 1 and 2, and the appropriate microplate was selected based on the STEC tested. In brief, one drop of either Shiga toxin 1 or Shiga toxin 2 conjugate was added to each microplate assay well. The overnight-enriched broths were diluted appropriately in the diluent provided, and 100 μL of each diluted sample was added to the designated microplate well. The microplate was sealed and incubated at 37 °C for 50 min. After incubation, the wells were washed with wash buffer, and the substrate was added. After incubating for 10 min at room temperature, plates were read at 450/620 nm within 10 min after adding the stop solution. In each microplate, separate wells were designated for the Shiga toxin standards (for developing the standard curve) and the positive and negative controls.

### 5.5. Determination of the Optimal Mitomycin C Concentration

A single colony from each STEC strain was inoculated into TSB and incubated at 37 °C for ~3–4 h until the OD value reached ~0.5. From this neat culture, serial dilutions up to 10^−6^ were prepared in PBS. A 100 μL aliquot of the 10^−6^ PBS dilution of each STEC culture was inoculated into TSB and treated with mitomycin C solutions with concentrations ranging from 5 to 500 ng/mL in a final volume of 3 mL. Sterilized laboratory grade water was used for the non-treated control. The broth mixtures were then incubated at 37 °C overnight for enrichment. On the following day, each tube of broth mixture was subjected to real-time PCR and both EIA assays (SHIGA TOXIN QUIK CHEK^TM^ and RUO Quantitative Shiga toxin ELISA).

### 5.6. Application of the Optimal Mitomycin C Concentration to Clinical STEC Isolates

Fifty-three STEC isolates were tested for their response to mitomycin C as an enhancer to induce Shiga toxin production. STEC isolates were grown and treated with mitomycin C as described in Section 5.1, at the optimal concentration as determined in Section 5.5. After an overnight enrichment at 37 °C, real-time PCR analysis was performed, and the presence of toxin was assessed by qualitative EIA assay using SHIGA TOXIN QUIK CHEK^TM^ kits; toxin quantification was not performed.

### 5.7. Application of the Optimal Mitomycin C Concentration to STEC-Positive Clinical Stool Samples

Aliquots of 15 patients’ stool samples submitted to ProvLab that tested positive for STEC by PCR and culture were collected and stored at 4 °C until used. A 10% stool suspension in PBS was prepared from each stool sample. Both TSB and GNB (BBL™ GN Broth, Becton, Dickinson and Company, Sparks, MD, USA) were used as the enrichment media, as frontline laboratories use both these media for stool culture. A 500 μL aliquot of each stool suspension was added to either broth with 100 μL of mitomycin C solution at the optimized concentration as determined in Section 5.5 to a final volume of 3 mL (100 μL of water was used for a non-treated control). After an overnight enrichment at 37 °C, each enriched broth was subjected to real-time PCR analysis and both EIA assays.

### 5.8. Statistical Analysis

Experiments to determine the optimum mitomycin C concentration were performed in triplicate on 3 separate days, and all analyses were performed in triplicate (3 biological replicates). PCR analysis and EIAs on the 53 STEC isolates and STEC-positive stool samples treated with and without mitomycin C were performed once. Each sample was analyzed in duplicate by PCR (2 technical replicates) and RUO Quantitative Shiga toxin ELISA. The antibiotic treatment effect was analyzed using one-way analysis of variance, and the treatments were compared using Bonferroni post-test. Treatment effects were considered statistically significant when *p* < 0.05. Data are presented as mean ± STDEV.

## Figures and Tables

**Table 1:** Induction of Shiga toxin production by different concentrations of mitomycin C after overnight enrichment in TSB.

STEC Isolate	*stx* Subtype	Mitomycin C Conc. (ng/mL)	Avg Ct ± STDEV	Avg Toxin Conc. ± STDEV (ng/mL)	SHIGA TOXIN QUIK CHEK^TM^
O5:H19	*stx1c*	0	13.82 ± 0.41	0.37 ± 0.03	+
		5	13.70 ± 0.18	0.84 ± 0.07	+
		10	13.81 ± 0.31	1.32 ± 0.08	+
		25	13.62 ± 0.19	2.30 ± 0.17	+
		50	13.31 ± 0.23	5.58 ± 0.92	+
		100	12.84 ± 0.11	13.81 ± 1.53	+
		500	13.17 ± 0.32	45.50 ± 4.84	+
O26:H11	*stx2a*	0	11.49 ± 0.08	21.76 ± 2.30	+
		5	11.03 ± 0.09	48.70 ± 17.07	+
		10	10.49 ± 0.11	90.88 ± 27.26	+
		25	9.67 ± 0.26	233.10 ± 92.74	+
		50	8.62 ± 0.07	698.94 ± 232.98	+
		100	7.66 ± 0.10	1947.25 ± 481.40	+
		500	9.40 ± 0.66	2582.43 ± 706.71	+
O157:H7	*stx1a*	0	11.64 ± 0.46	10.62 ± 0.03	+
*(stx1/2)*		5	11.19 ± 0.24	10.96 ± 0.36	+
		10	10.78 ± 0.14	12.48 ± 0.36	+
		25	10.36 ± 0.07	19.89 ± 3.51	+
		50	9.94 ± 0.18	22.26 ± 1.50	+
		100	9.67 ± 0.11	29.51 ± 2.81	+
		500	9.95 ± 0.23	45.93 ± 1.38	+
	*stx2a*	0	11.59 ± 0.09	17.92 ± 1.24	+
		5	11.16 ± 0.12	41.87 ±1.72	+
		10	10.86 ± 0.16	58.10 ± 1.97	+
		25	10.14 ± 0.12	106.68 ± 4.79	+
		50	9.52 ± 0.10	159.82 ± 1.56	+
		100	9.34 ± 0.04	204.49 ± 8.74	+
		500	9.34 ± 0.07	237.31 ±7.60	+

Experiments and analyses were performed in triplicate on three individual days. Data reported as mean ± STDEV. ‘+’: Positive.

**Table 2:** Shiga toxin production by 53 STEC isolates with and without mitomycin C treatment after overnight enrichment in TSB.

Organism	Subtype	SHIGA TOXIN QUIK CHEK^TM^				PCR (Ct)			
		NT		MC		NT		MC	
		*stx1*	*stx2*	*stx1*	*stx2*	*stx1*	*stx2*	*stx1*	*stx2*
O118:H2	*stx1a*	+		+		12.14		12.81	
O118:H16/O151	*stx1a*	+		+		11.69		11.71	
O118/O151:H16	*stx1a*	+		+		11.92		11.31	
O118/O151:H2	*stx1a*	+		+		11.11		12.07	
O121:H19	*stx1a*	+		+		11.58		11.34	
O121:H19	*stx1a*	+		+		11.94		10.84	
O121:H19	*stx2a*		+		+		12.46		11.90
O111:H8	*stx1a*	+		+		11.12		12.31	
O111:H Non-motile	*stx1a*	+		+		12.19		12.79	
O111:H Non-motile	*stx1a*, *stx2a*	+	+	+	+	11.44	11.92	11.34	11.21
O123/O186:H2	*stx1a*	+		+		12.09		12.03	
O123/O186:H2	*stx1a*	+		+		11.68		11.12	
O123/O186:H2	*stx1a*, *stx2a*	+	+	+	+	11.48	11.97	11.61	12.11
O146:H21	*stx1c*	+		+		13.64		13.69	
O146:H21	*stx1c*, *stx2b*	+	+	+	+	13.15	12.49	13.06	12.30
O146:H21	*stx1c*, *stx2b*	+	-	+	+	13.48	13.09	13.73	13.19
O26:H11	*stx1a*	+		+		11.91		12.35	
O26:H11	*stx2a*		-		-		11.90		9.65
O26:H11	*stx1a*, *stx2a*	+	+	+	+	11.66	11.68	11.44	11.56
O103:H2	*stx1a*	+		+		11.82		12.11	
O103:H25	*stx1a*	+		+		12.05		12.14	
O108:H21	*stx1c*	-		+		13.34		13.37	
O108:H2	*stx2a*, *stx2d*		+		+		12.40		9.85
O145:H Non-motile	*stx1a*	+		+		11.89		11.67	
O145:H7	*stx2a*		+		+		11.29		10.87
O156:H25	*stx1a*	+		+		11.64		11.97	
O156:H14	*stx2f/h/b*		-		-		15.21		15.71
O157:H7	*stx2a*		-		-		11.90		10.88
O157:H7	*stx1a*, *stx2c*	+	+	+	+	11.79	12.25	11.89	12.51
O166:H15	*stx2f/h/b*		+ *		+ *		13.83		13.70
O166:H15	*stx2h*		-		+ *		14.37		13.59
O177:H11	*stx1a*	+		+		11.51		11.50	
O177:H25	*stx2c*		+		+		12.65		12.14
O5:H9	*stx1a*	+		+		10.02		10.39	
O5:H9	*stx1a*, *stx2a*	+	+	+	+	10.95	11.46	11.35	11.51
O71:H8	*stx1*	+		+		13.00		12.31	
O71:H8	*stx1a*, *stx2c*	+	+	+	+	12.33	12.31	12.96	11.35
O91:H14	*stx2b*		+		+		12.91		12.56
O91:H14	*stx1*, *stx2b*	+	+	+	+	11.24	11.59	16.20	15.32
O98:H Non-motile	*stx1a*	+		+		11.73		12.11	
O98:H Non-motile	*stx1a*, *stx2a*	+	+	+	+	11.48	11.73	11.48	10.48
O85:H1	*stx2f/h/b*		+ *		+ *		13.14		13.75
O85:H2	*stx1a*	+		+		11.77		11.25	
O69:H11	*stx1a*	+		+		13.34		12.80	
O84:H Non-motile	*stx1a*	+		+		12.14		11.71	
O140:H21	*stx2c*		+ *		+ *		10.92		10.84
O17/O77/O44/O106:H45	*stx2d*		-		-		12.31		12.56
O113:H4	*stx2d*		+		+		11.44		10.91
O25:H1	*stx2e*		-		+		12.95		11.43
O2/O50:H7	*stx2f/h/b*		-		-		13.44		14.33
O75:H8	*stx1c*, *stx2b*	+	+	+	+	12.74	12.39	12.79	11.43
O112:H21	*stx1c*, *stx2b*	+	+	+	+	13.53	13.14	13.78	12.78
O128:H2	*stx1c*, *stx2b*	+	+	+	+	13.50	12.91	13.42	12.60
O175:H27(negative control)	-	ND	ND	ND	ND	ND	ND	ND	ND

* Very faint bands. NT: Non-treated, MC: Mitomycin C-treated. STEC serotypes with different strains are grouped. ‘+’: Positive, ‘-’: Negative, ND: Not detected.

**Table 3:** Real-time PCR results for 15 STEC-positive stool samples after overnight enrichment in TSB and GNB with and without mitomycin C.

ID	STEC Serotypes	*stx* Subtype	PCR (Ct)											
			*stx1*						*stx2*					
			TSB		GNB		* ∆Ct_TSB-GNB_		TSB		GNB		* ∆Ct_TSB-GNB_	
			NT	MC	NT	MC	NT	MC	NT	MC	NT	MC	NT	MC
1	O157:H7	*stx2a*, *stx2c*							16.1	15.9	16.9	15.4	−0.8	0.5
2		*stx1a*, *stx2a*	12.6	12.6	15.2	13.4	−2.6	−0.8	13.1	11.1	15.9	12.8	−2.9	−1.8
3		*stx1a*, *stx2a*	16.4	15.8	16.6	15.7	−0.2	0.1	16.8	16.3	16.7	16.1	0.0	0.1
4		*stx1a*, *stx2a*	15.0	14.1	15.0	13.2	0	0.9	15.3	14.4	15.2	13.5	0.0	0.9
5	O26:H11	*stx1a*	18.8	18.9	23.1	23.4	−4.3	−4.5						
6		*stx1a*	20.8	20.4	18.5	18.8	2.3	1.6						
7	O103:H2	*stx1a*	15.4	14.5	14.6	15.0	0.8	−0.5						
8		*stx1a*	25.1	23.1	20.8	19.7	4.3	3.4						
9	O151/O118:H16	*stx1a*	17.3	18.0	16.8	17.2	0.5	0.8						
10		*stx1a*	26.7	26.4	23.5	23.5	3.2	2.9						
11	O91:H14	*stx1a*	15.3	14.2	13.7	14.4	1.6	−0.2						
12	O121:H19	*stx2a*							18.0	18.0	16.9	15.8	1.1	2.2
13	O9:H7	*stx2a*							13.6	13.5	12.6	12.5	1.1	0.9
14	O146:H21	*stx1c*, *stx2b*	16.3	16.0	14.0	14.1	2.3	1.9	15.6	15.4	13.9	13.2	1.7	2.1
15	O128:H2/O5:H9	*stx1a*, *stx1c*, *stx2b*	15.4	14.7	13.9	15.2	1.5	−0.5	15.0	14.3	13.9	14.6	1.1	−0.3

NT: non-treated, MC: mitomycin C, TSB: trypticase soy broth, GNB: Gram-negative broth. * Interpretations_:_ Positive ∆Ct_TSB-GNB_: GNB improved STEC growth, Negative ∆Ct_TSB-GNB_: TSB improved STEC growth.

**Table 4:** SHIGA TOXIN QUIK CHEK^TM^ and STEC-ELISA results for 15 STEC-positive stool samples in TSB and GNB with and without mitomycin C treatment after overnight enrichment.

ID	Serotype	*stx* Subtype	*stx1*								*stx2*							
			SHIGA TOXIN QUIK CHEK^TM^				Toxin Conc. (ng/mL Toxin)				SHIGA TOXIN QUIK CHEK^TM^				Toxin Conc. (ng/mL Toxin)			
			TSB		GNB		TSB		GNB		TSB		GNB		TSB		GNB	
			NT	MC	NT	MC	NT	MC	NT	MC	NT	MC	NT	MC	NT	MC	NT	MC
1	O157:H7	*stx2a*, *stx2c*									+	+	+	+	1.50	9.06	3.90	15.71
2		*stx1a*, *stx2a*	+	+	+	+	1.62	6.51	4.06	7.83	-	+	+	+	ND	137.79	11.01	582.76
3		*stx1a*, *stx2a*	+	+	+	+	1.36	1.11	1.72	1.94	+	+	+	+	0.81	5.05	0.83	8.41
4		*stx1a*, *stx2a*	+	+	+	+	0.62	1.59	0.97	4.42	+	+	+	+	0.69	14.27	1.71	12.99
5	O26:H11	*stx1a*	-	-	-	-	0.19	0.20	ND	ND								
6		*stx1a*	-	-	-	+	ND	ND	ND	0.21								
7	O103:H2	*stx1a*	-	+	-	+	ND	ND	ND	0.19								
8		*stx1a*	+	+	+	+	0.20	0.34	0.22	1.46								
9	O151/O118:H16	*stx1a*	-	+	+	+	0.23	0.30	0.39	0.44								
10		*stx1a*	-	-	-	-	ND	ND	ND	ND								
11	O91:H14	*stx1a*	+	+	-	+	0.36	0.42	0.45	0.49								
12	O121:H19	*stx2a*									+	+	+	+	0.56	3.05	2.72	12.92
13	O9:H7	*stx2a*									-	-	-	-	ND	ND	ND	ND
14	O146:H21	*stx1c*, *stx2b*	-	+	+	+	0.11	0.44	0.33	1.08	-	+	+	+	ND	2.86	ND	15.96
15	O128:H2/O5:H9	*stx1a*, *stx1c*, *stx2b*	-	-	-	-	0.10	0.22	0.12	ND	-	+	-	+	ND	1.98	ND	5.77

NT: non-treated, MC: mitomycin C, TSB: trypticase soy broth, GNB: Gram-negative broth, ND: not detected. ‘+’: Positive, ‘-’: Negative.

**Table 5:** Primer and probe sequences used in this study *.

Reference Gene/Primer/Probe	Sequence 5′-3′
*stx1*-Forward	TTT GTY ACT GTS ACA GCW GAA GCY TTA CG
*stx1*-Reverse	CCC CAG TTC ARWGTR AGR TCM ACR TC
*stx1*-Probe	FAM/CTGGATGAT/ZEN/CTCAGTGGGCGTTCTTATGTAA/IABkFQ
*stx2*- Forward	TTT GTY ACT GTS ACA GCW GAA GCY TTA CG
*stx2*-Reverse	CCC CAG TTC ARWGTR AGR TCM ACR TC
*stx2*-Probe	YAkYel/TCGTCAGGC/ZEN/ACTGTCTGAAACTGCTCC/IABkFQ

* The primers and probe sequences were adapted from Perelle et al. 2004 [23]. Y is (C, T), S is (C, G), W is (A, T), R is (A, G), M is (A, C).

## Data Availability

The original contributions presented in this study are included in this article and Supplementary Material. Further inquiries can be directed to the corresponding author.

## Supplementary Materials

The following supporting information can be downloaded at: https://www.mdpi.com/article/10.3390/toxins17060267/s1, Figure S1: *SHIGA TOXIN QUIK CHEK*^TM^ images of three STEC isolates with *stx1*, *stx2*, and *stx1 and 2* incubated overnight in TSB with different concentrations of mitomycin C.; Figure S2: *SHIGA TOXIN QUIK CHEK*^TM^ images of STEC isolates enriched in TSB overnight with 500 ng/mL mitomycin C in TSB.; Figure S3: *SHIGA TOXIN QUIK CHEK*^TM^ images showing STEC-positive stool samples enriched overnight in TSB and GNB with and without 500 ng/mL mitomycin C.

## Abbreviations

The following abbreviations are used in this manuscript:

EIA	Enzymatic immune assay
GNB	Gram-negative broth
HUS	Hemolytic uremic syndrome
PCR	Polymerase chain reaction
RUO	Research use only
STEC	Shiga toxin-producing Escherichia coli
TSB	Trypticase soy broth
