# Supplementary material for: The Effect of Mitomycin C on Induction of Shiga Toxin Production in Clinical STEC Isolates
Source: Toxins (Basel). 2025 May 27;17(6):267. doi: 10.3390/toxins17060267 (PMC12197360; doi:10.3390/toxins17060267)
Supplement: Supplementary file 1 [file toxins-17-00267-s001.zip › toxins-3643532-supplementary.pdf]

## Suppelementary Figures

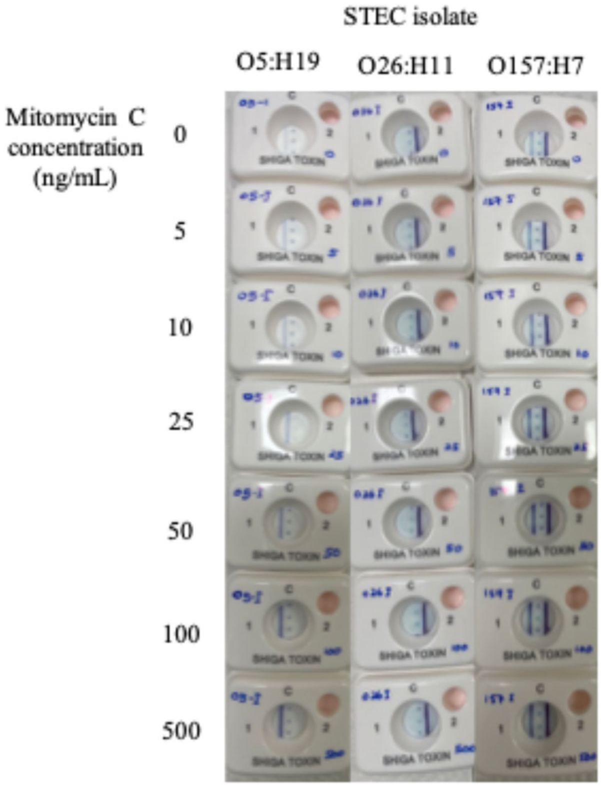


**Figure S1**: *SHIGA TOXIN QUIK CHEK*™ images of 3 STEC isolates with s*tx1*, *stx2*, and *stx1&2* incubated overnight in TSB with different concentrations of mitomycin C.

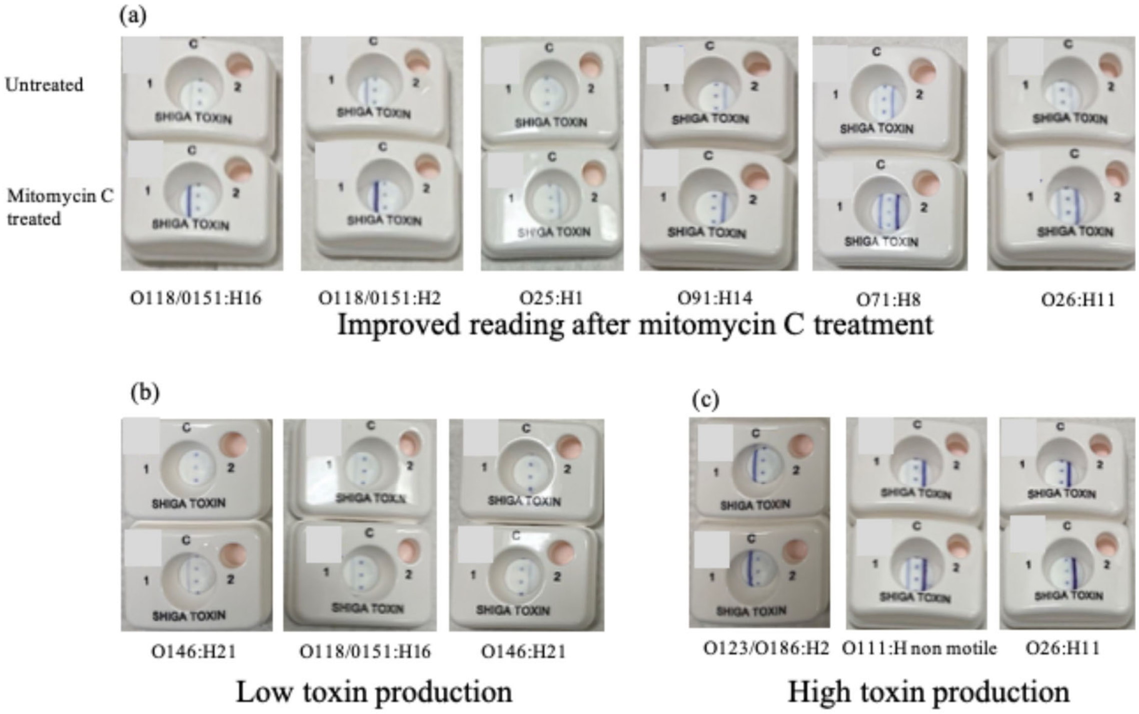


**Figure S2:** *SHIGA TOXIN QUIK CHEK*™ images of STEC isolates enriched in TSB overnight with 500 ng/mL mitomycin C in TSB. (a) *SHIGA TOXIN QUIK CHEK*™ images that show improved reading after the mitomycin C treatment. (b) Images that showed faint bands pointing to low toxin production. (c) Images that showed thicker bands pointing to high toxin production.

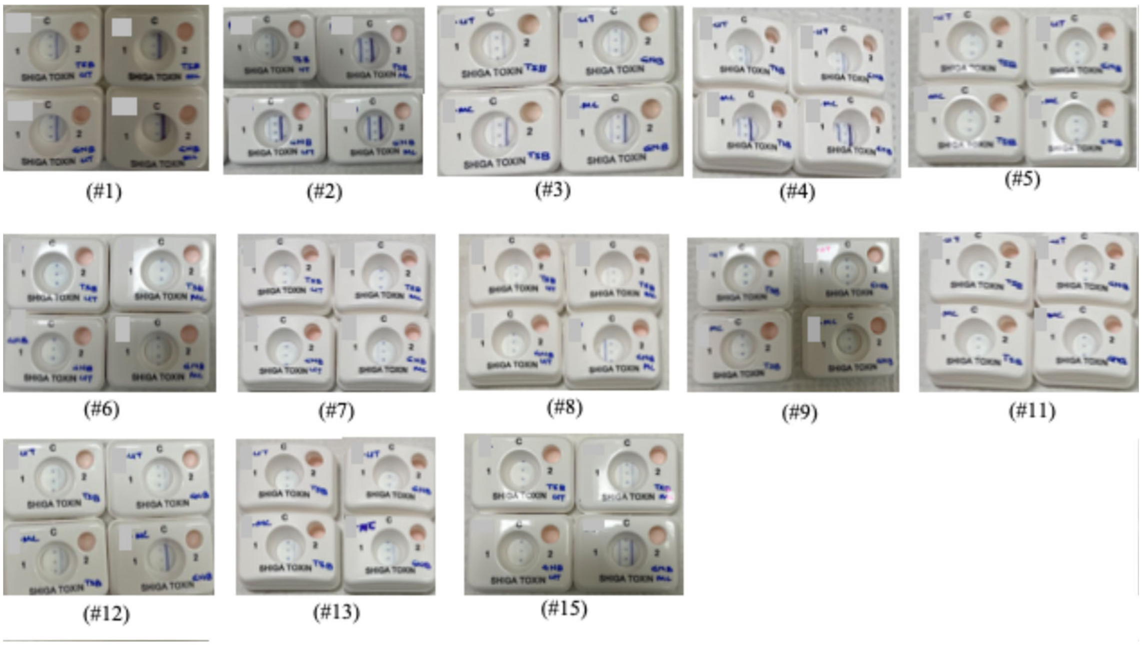


**Figure S3:** *SHIGA TOXIN QUIK CHEK™* images showing STEC positive stools enriched overnight in TSB and GNB with and without 500 ng/mL mitomycin C. The ID numbers on the image corresponds to the Tables 4 and 5.
